# Transcriptomics Analysis on Excellent Meat Quality Traits of Skeletal Muscles of the Chinese Indigenous Min Pig Compared with the Large White Breed

**DOI:** 10.3390/ijms19010021

**Published:** 2017-12-22

**Authors:** Yingzi Liu, Xiuqin Yang, Xiaoyan Jing, Xinmiao He, Liang Wang, Yang Liu, Di Liu

**Affiliations:** 1 College of Animal Sciences and Technology, Northeast Agricultural University, Harbin 150030, China; yingziliu1229@gmail.com(Y.L.); xiuqinyang@neau.edu.cn (X.Y.); xiaoyanjing373@163.com(X.J.); 18724617749@163.com(Y.L.); 2 Heilongjiang Academy of Agricultural Sciences, Harbin 150086, China; haashxm@163.com (X.H.); wlwl448@163.com (L.W.)

**Keywords:** Min pig, transcriptomics, meat quality, *longissimus dorsi*, *biceps femoris*

## Abstract

The Min pig (*Sus scrofa*) is a well-known indigenous breed in China. One of its main advantages over European breeds is its high meat quality. Additionally, different cuts of pig also show some different traits of meat quality. To explore the underlying mechanism responsible for the differences of meat quality between different breeds or cuts, the *longissimus dorsi* muscle (LM) and the *biceps femoris* muscle (BF) from Min and Large White pigs were investigated using transcriptome analysis. The gene expression profiling identified 1371 differentially expressed genes (DEGs) between LM muscles from Min and Large White pigs, and 114 DEGs between LM and BF muscles from the same Min pigs. Gene Ontology (GO) enrichment of biological functions and Kyoto Encyclopedia of Genes and Genomes (KEGG) analysis showed that the gene products were mainly involved in the IRS1/Akt/FoxO1 signaling pathway, adenosine 5′-monophosphate-activated protein kinase (AMPK) cascade effects, lipid metabolism and amino acid metabolism pathway. Such pathways contributed to fatty acid metabolism, intramuscular fat deposition, and skeletal muscle growth in Min pig. These results give an insight into the mechanisms underlying the formation of skeletal muscle and provide candidate genes for improving meat quality. It will contribute to improving meat quality of pigs through molecular breeding.

## 1. Introduction

Pigs are an important source of meat production worldwide [1]. To meet the increasing quality demands, it is necessary to improve the quality of pork. Pork quality is influenced by many factors, including breed, nutrition, production system and post-slaughter handling [2]. Among these factors, breed is the most significant. In China, there are more pig breeds than in any other country all over the world [3]. There are 118 indigenous pig breeds in China according to the Domestic Animal Diversity in the World index [4]. Previously, breeders have aimed to increase muscle yield and decrease carcass fatness, which caused great progress for these characters in swine breeding. For example, Large White (LW) and Landrace pigs have the traits of high growth and lean meat percentage. Recently, some studies suggest that the process of such intensive selection have led to a deterioration in meat quality [5,6,7]. 

As a typical lean-type European breed, the LW pig is now widely used for commercial production [8]. Compared with European breeds, Chinese indigenous pig breeds have higher intramuscular fat (IMF), increased tenderness and better meat quality [9,10,11]. The Min pig, which only exists in the northeast of China, such as Heilongjiang Province, is a well-known Chinese fat-type breed. It is famous for its significant characteristics, such as high IMF, superior meat quality, and strong resistance to crude feed, general diseases and cold. Compared with Min, the LW pig has a faster growth rate and higher lean meat ratio. Therefore, these two breeds can serve as an ideal comparison for studying differences in meat quality between European commercial pigs and Chinese indigenous pigs [8].

Different cuts of pork are made into kinds of meat products to meet varied demands of customers. There are differences in meat quality among different cuts of the same individual [12,13]. *Longissimus dorsi* (LM) and *biceps femoris* (BF) are both major sources of raw materials for meat products, which have a large share of the market, and showing different characteristics in meat quality. However, the mechanism of the difference is still not fully understood. 

Skeletal muscle consists of different fiber types which are characterized by the myosin heavy chain (MyHC) isoform composition. There are up to four main fiber types: I, IIa, IIx and IIb [14]. One of the main factors determining muscle biochemical pathways is fiber type composition, which results from the coordinated expression of distinct sets of structural protein and metabolic enzymes. The skeletal muscle fiber number, size, and fiber type composition were closely related to each other, and there was a correlation between skeletal muscle fiber characteristics and meat quality traits [15]. The skeletal muscle fiber type transformation can be regulated by different signaling pathways and regulatory factors in vivo. For example, the insulin signaling pathway has been found to play a role in the skeletal muscle fiber type transformation [16]. In addition, the forkhead box O1 (FoxO1) was reported to negatively regulate MyHC I formation [17,18]. It is important to find out the underlying signaling pathways and potential regulatory factors to improve meat quality. 

IMF content is also an important factor of meat quality, which has a positive correlation with meat tenderness and juiciness, and could be affected by breeds and nutritional regulation. Moderate IMF ensures a pleasant eating experience while lean meat often associates with tough texture [19]. It has been reported that the IMF of European pigs is 2% to 3%, while in Chinese indigenous pigs it is always 4%, and in Min pig it could achieve 5%. Some studies suggest that high quality pork contains high IMF [20,21]. Thus, an effective means to increase meat quality is increasing IMF, which impels us to find out the underlying mechanism. Adenosine 5′-monophosphate-activated protein kinase (AMPK) is a sensor of cellular energy status involved in regulation of glycogen metabolism and lipid metabolism. It influences meat quality by modulating fat content in skeletal muscle. The activity and cascade effects of AMPK are major regulating factors of meat quality [22].

In this study, we applied the transcriptomic technique to obtain differentially expressed genes (DEGs) between the same cuts of different breeds (LM of LW and Min), or different cuts of the same breed (LM and BF of Min). Gene Ontology (GO) terms and the Kyoto Encyclopedia of Genes and Genomes (KEGG) pathway analysis were adopted to characterize the expression profiles in the muscles of LW and Min. The aim of this study was to reveal the molecular mechanism underlying the difference of meat quality between different breeds or cuts on the transcriptome level.

## 2. Results

### 2.1. RNA Sequencing Data Mapping and Annotation

In total, 9 cDNA libraries from three groups (LW_LM, Min_LM, Min_BF, three replications for each group) were sequenced, which yielded 271 million 100 bp paired-end clean reads in total, varying from 30.0 to 30.4 million for each sample (Table 1). Among the clean reads, more than 97.36% had quality scores at the Q20 level, and on average, approximately 85.03% clean reads were mapped to the reference genome (*Sus scrofa* 11.1) (Table 1).

**Figure 1:**
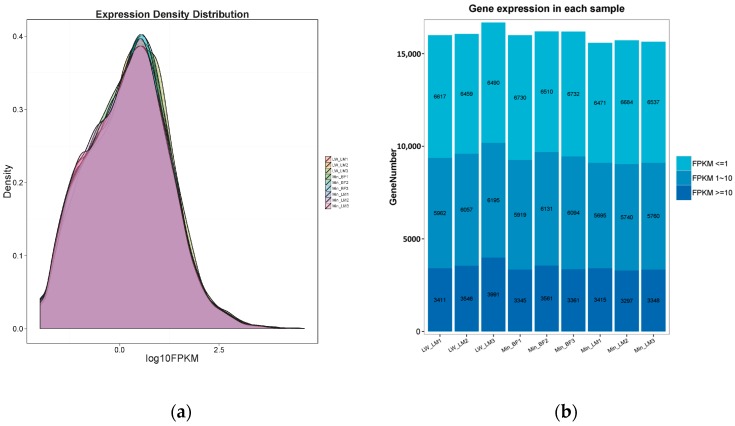
(**a**) The density plot of genes’ log10 (FPKM) distribution visualized by CummeRbund. The *X*-axis represents the log10 (FPKM) of all the genes. The *Y*-axis represents the genes’ distribution density. The nine groups were shown by different colors; (**b**) The histogram of gene expression. The *X*-axis represents individual sample. The *Y*-axis represents the number of expressed genes. The color depth represents the expression level of genes.

After assembling for each sample, Cuffdiff package in Cufflinks (available online: http://coletrapnell-lab.github.io/cufflinks/), a program aiming to find significant changes in expression levels of genes and transcripts in RNA-Seq experiments, was used to calculate the expected number of fragments per kilobase of transcript sequence per millions base pairs sequenced (FPKM) of the three groups for each gene according to the pig 11.1 reference genome annotation. With the FPKM threshold of 1 and 10, a statistical result was shown in Figure 1a, respectively. As shown in Figure 1b, the FPKM density of nine pigs displayed similar skewed distribution and approximately 20.76–23.93% genes were found to be lowly expressed (FPKM ≤ 1) (Figure 1b).

### 2.2. Differentially Expressed Genes Calling and Validation by Real-Time PCR

**Figure 2:**
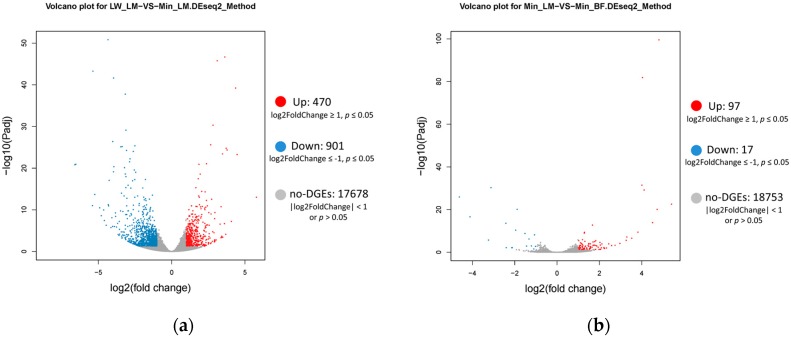
Volcano plot for differentially expressed genes (DEGs). The *X*-axis represents the log2 (FPKM) of all the genes. The *Y*-axis represents –log10 (*p* value). Red represents up-regulate DEGs. Blue represents down-regulate DEGs. Grey represents no-DEGs. (**a**) DEGs of Group LM; (**b**) DEGs of Group Min.

**Figure 3:**
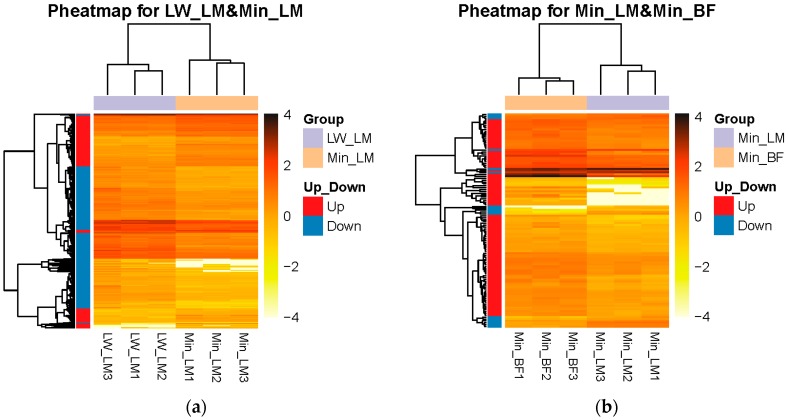
Pheatmaps for DEGs. The *X*-axis represents samples. The *Y*-axis represents DEGs. The color depth represents –log10 (FPKM). Red represents up-regulate DEGs. Blue represents down-regulate DEGs. (**a**) DEGs of Group LM; (**b**) DEGs of Group Min.

To quantify the basic genetic difference between LW and Min pigs, or LM and BF muscles, we analyzed the transcriptome difference in the Group LM (LW_LM vs. Min_LM, LW_LM as control) and the Group Min (Min_LM vs. Min_BF, Min_LM as control), respectively. Compared with LW_LM, 470 DEGs expressed more and 901 DEGs expressed less in Min_LM (*p* < 0.05 and |log2FoldChange| ≥ 1) in the Group LM (Figure 2a). In the Group Min, compared with Min_LM, we got 97 DEGs more expressed and 17 DEGs less expressed in Min_BF (Figure 2b). The heatmap for DEGs of two Groups showed that associated with the three parts of muscles, three clusters have three distinct gene expression patterns (Figure 3a,b).

**Figure 4:**
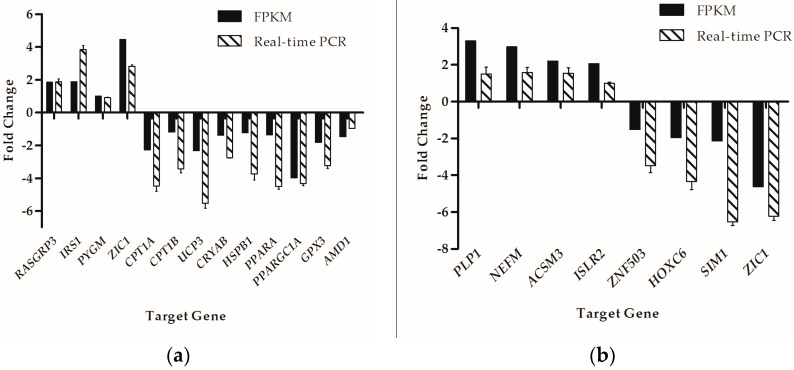
Real-time PCR validation of the DEGs analyzed by RNA-seq. (**a**) 13 genes that were identified as DEGs in Group LM; (**b**) 8 genes that were identified as DEGs in Group Min. The *Y*-axis shows the relative expression levels.

To validate the expression pattern of DEGs identified from RNA-Seq, 21 genes were selected in total to perform the real-time PCR assays with the *GAPDH* gene as internal control. Among these genes, 13 genes were selected for Group LM, while the other 8 genes were for Group Min. The real-time PCR results were consistent with the RNA-Seq results as shown in Figure 4.

### 2.3. Gene Ontology Analysis and Kyoto Encyclopedia of Genes and Genomes Pathway Analysis of DEGs

**Figure 5:**
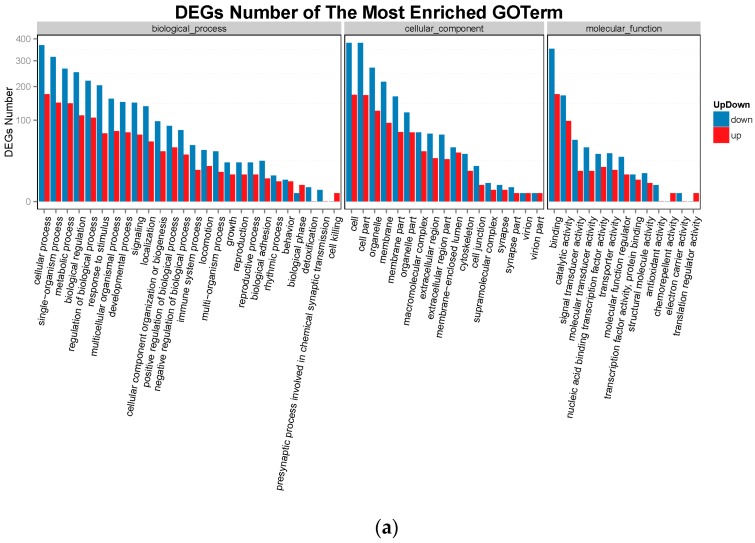

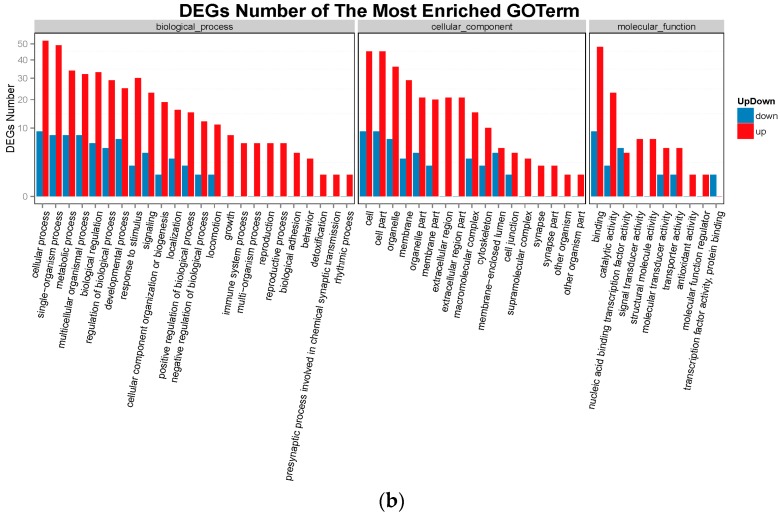
The column diagrams for Gene Ontology (GO) analysis of DEGs. The *X*-axis represents the functions of GO analysis. The *Y*-axis represents the numbers of DEGs. Red represents up-regulate DEGs. Blue represents down-regulate DEGs. (**a**) DEGs of Group LM; (**b**) DEGs of Group Min.

To further determine the functions of the DEGs, functional categorization of all the DEGs was performed using GO annotation. The annotated results were classified into three parts: biological process, cellular component and molecular function (Figure 5). The top 5 of each part for Group LM were shown as: (1) biological process: (i) cellular process; (ii) single-organism process; (iii) metabolic process; (iv) biological regulation; (v) regulation of biological process; (2) cellular component: (i) cell; (ii) cell part; (iii) organelle; (iv) membrane; (v) membrane part; (3) molecular function: (i) binding; (ii) catalytic activity; (iii) signal transducer activity; (iv) molecular transducer activity; (v) nucleic acid binding transcription factor activity. The top 5 of each part for Group Min were shown as: (1) biological process: (i) cellular process; (ii) single-organism process; (iii) metabolic process; (iv) multicellular organismal process; (v) biological regulation; (2) cellular component: (i) cell; (ii) cell part; (iii) organelle; (iv) membrane; (v) organelle part; (3) molecular function: (i) binding; (ii) catalytic activity; (iii) nucleic acid binding transcription factor activity; (iv) signal transducer activity; (v) structural molecule activity.

**Figure 6:**
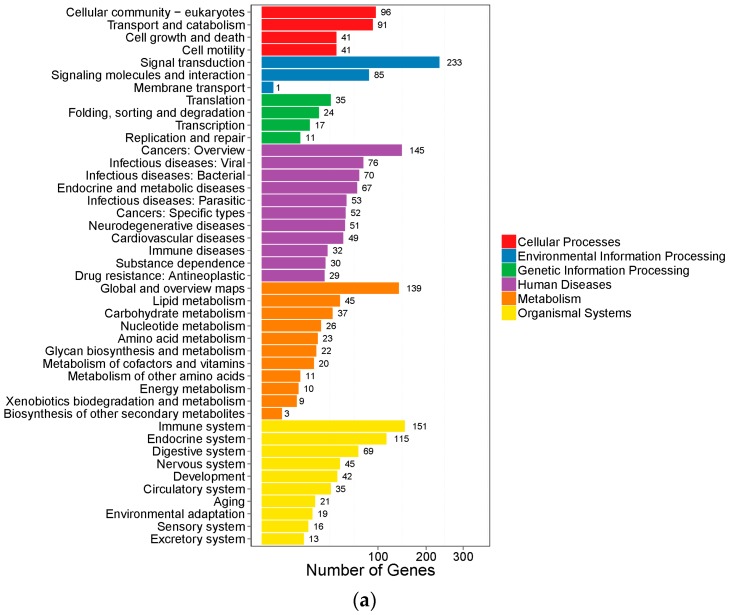

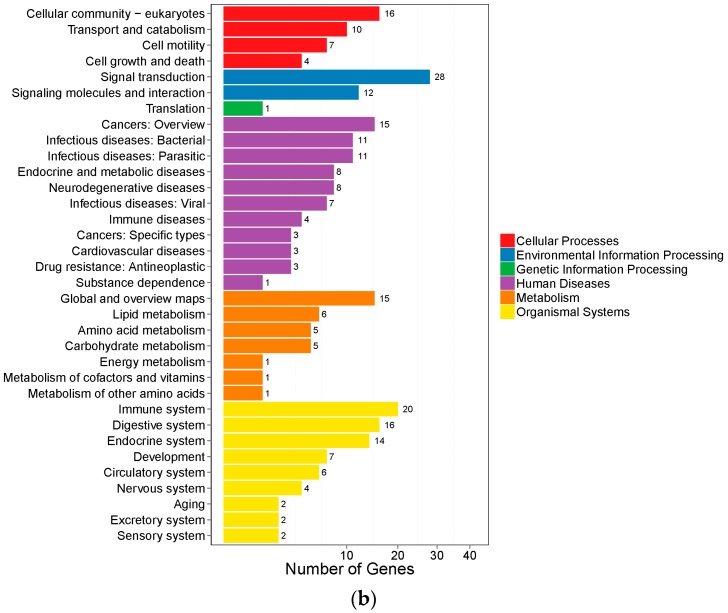
The column diagrams for Kyoto Encyclopedia of Genes and Genomes (KEGG) analysis of DEGs. The *X*-axis represents the numbers of DEGs. The *Y*-axis represents the functions of pathways. Each color represents the appropriate biological process. (**a**) DEGs of Group LM; (**b**) DEGs of Group Min.

**Figure 7:**
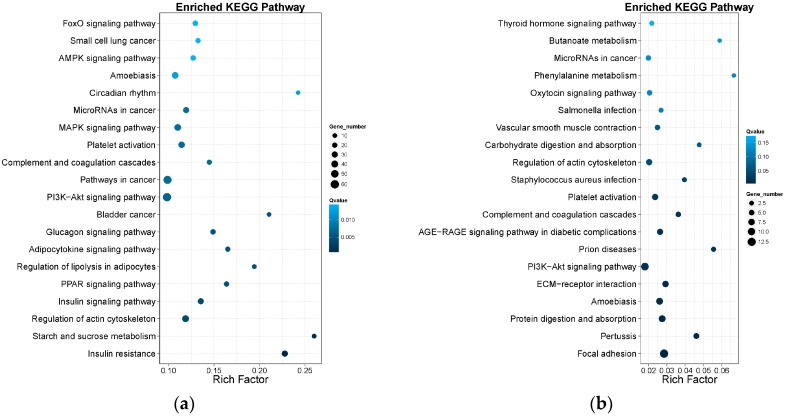
The diagrams for the enrichment degree of pathways. The *X*-axis represents the value of rich factors (the ratio of annotated DEGs to all genes of enriched pathway). The *Y*-axis represents the names of pathways. The color depth of each point represents *q* value. The size of each point represents the number of DEGs. (**a**) Enrichment degree of Group LM; (**b**) Enrichment degree of Group Min.

The KEGG analysis of DEGs was also performed. As shown in Figure 6, pathways were classified in six classifications by the function: cellular processes, environmental information processing, genetic information processing, human diseases, metabolism and organismal systems. At the same time, the pathways were also sorted by correlativity of enrichment (Figure 7). We got 63 pathways (*p* < 0.05) in Group LM, of which the top 30 pathways were shown in Table 2. All the 28 pathways (*p* < 0.05) of Group Min were shown in Table 3. 

## 3. Discussion

### 3.1. IRS1/Akt/FoxO1 Signaling Pathway

The insulin signaling pathway appears to play a large role in not only the metabolism of carbohydrate and lipid, but also the development and growth of muscle. Most of the metabolic effects of insulin are mediated by the signaling pathway involving the phosphorylation of the insulin receptor substrate 1 (IRS1), and the activation of the phosphatidylinositol 3-kinase (PI3K), protein kinase B (Akt), and FoxO1 [16,23]. 

Recently, the IRS1/Akt/FoxO1 signaling pathway was reported to appear in sarcopenia. IRS1 works as a molecular switch in the insulin signaling pathway, and activates the PI3K signal transduction pathway under insulin stimulation. In knockout mice, insulin responses and skeletal muscle masses were slightly affected by IRS1. Akt is the downstream factor of IRS1, and it is also the key intersection of the muscular atrophy pathway and muscular hypertrophy pathway. Activated Akt inhibits atrophy and activates hypertrophy [24]. FoxO1 is the downstream factor of Akt and negatively regulates muscle mass [25]. It has been reported that, in the galactose injection induced aged mice model, low activated IRS1 or Akt and high activated FoxO1 associated with muscular atrophy could be improved by exercise remission, through the way of activated IRS1 and Akt, facilitated by the phosphorylation of FoxO1 [26].

Compared with LW_LM, we got higher expressed IRS1, lower expressed FoxO1, and higher expressed Akt (whereas the difference of Akt was not significant) in Min_LM, which may result in higher muscle mass and better meat quality in Min pig. However, the level of their phosphorylation needs experimental verification, and the upstream factors could be further explored. 

FoxO1 also plays a critical role in skeletal muscle type specification. The study of Kamei et al. showed that transgenic mice specifically overexpressing FoxO1 in skeletal muscle weighed less than the wildtype control mice and showed significantly reduced muscle mass as well as the size of both MyHC I and MyHC II fibers. Meanwhile, the MyHC I fiber-related gene expression and the number of MyHC I fibers were decreased markedly [17]. It was also reported that in pig myoblasts, FoxO1 gene silence promoted the expression of MyHC I [18]. These findings appear to show that FoxO1 negatively regulate MyHC I formation. Compared with LW pig, Min had more MyHC I fibers and better meat quality. The lower expressed FoxO1 could be responsible for meat quality. FoxO1 may, in part, repress the MyHC I gene expression through regulating the activities of myocyte-specific enhancer factor 2C (MEF2C) or Ca^2+^/calmodulin-dependent protein kinase (CaMK) [27]. In addition, the activity of FoxO1 could be regulated by post transcriptional modifications such as phosphorylation, acetylation and ubiquitination. The certain mechanisms could be validated in following experiments.

### 3.2. AMPK Activity and Cascade Effects

AMPK is a serine-threonine kinase that functions primarily as a metabolic sensor to coordinate anabolic and catabolic activities in the cell via the phosphorylation of multiple proteins involved in metabolic pathways, such as lipid metabolism, glucose catabolism and protein synthesis. Color, pH value and Water Holding Capacity (WHC) were affected by activation or inhibition to PGC-1α and FoxO1, associated with glucose transporter 4 (GLUT4) activation or inhibition in glucose catabolism. Similarly, the regulation of sterol regulatory element-binding proteins (SREBPs) induced activity changing of acetyl CoA carboxylase (ACC) and fatty acid synthase (FAS) in lipid metabolism, affecting the flavor, tenderness, juiciness, color and WHC of meat [28]. 

In this study, AMPK was lower expressed in Min pig than LW. In the glucose catabolism pathway, PGC-1α and FoxO1 were both lower expressed but the downstream targets GLUT4 had no marked difference. It suggested that the effects of AMPK/PGC-1α or AMPK/FoxO1 signaling may affect meat quality through other process like muscle fiber type changes, but not glucose catabolism. In the pathway of lipid metabolism, we got higher expressed sterol regulatory element-binding protein 1c (SREBP1c) and lower expressed hormone-sensitive triglyceride lipase (HSL) in Min pig. The former had a positive correlation with fatty acid synthesis [29], while the latter also appeared in regulation of lipolysis in the adipocytes pathway. HSL was considered to be the key rate-limiting enzyme responsible for regulating triacylglycerol (TAG) mobilization [30]. Low level expressed HSL could decrease lipolysis and lead to fat deposition in Min. Meanwhile, CGI-58, MGL and aFABP were all lower expressed in Min pig, which could decrease lipolysis and contribute to fat deposition. 

Regulation of the biological effect by AMPK has the benefit to improve meat quality because of the varied functions of AMPK and its cascade effects. However, the underlying molecular mechanism is still unclear and warrants further investigation. 

### 3.3. DEGs of Lipid Metabolism

According to the pathway analysis, lipid metabolism consists of ketogenesis, lipid transport, lipogenesis, cholesterol metabolism, fatty acid transport and fatty acid oxidation. Several DEGs appeared in these biological processes which led to the differences of IMF and influenced meat quality. 

The liver X receptor α (LXRα) plays a leading role in lipid and glucose metabolism, which was higher expressed in Min_LM compared with LW_LM. LXRα increases fatty acid oxidation, decreases serum triglyceride and improves glucose metabolism. Zheng W. had reported that in RAW264.7 cells, total cholesterol and free cholesterol were both down-regulated by curcumine cure associated with LXRα up-regulation [31]. Su [32] and Gong [33] had reported that the expression level of LXRα showed a positive correlation with lipid deposition. In this study, the high expression of LXRα may lead to high IMF in Min pig and contribute to improved meat quality. 

In the fatty acid transport pathway, fatty acid binding protein 3 (FABP3), lipoprotein lipase (LPL), and long-chain acyl-CoA synthetase (ACSL) were all low expressed in Min_LM. FABP3 was regarded as candidate genes for carcass fatness traits in pigs. The fat content of whole carcass and primary cuts was positively and highly (*p* < 0.01) correlated with the mRNA abundance of FABP3 gene. Furthermore, the expression of the FABP3 gene showed a significantly (*p* < 0.001) higher level in BF compared to LM [34]. It was also reported that the mRNA abundance of FABP3 gene had no remarkable association with IMF between different breeds in bovine. In this study, FABP3 expressed lower in Min_LM than in LW_LM, but did not differ significantly between Min_LM and Min_BF, although the IMF of Min was significantly higher than that in LW. LPL is the rate-limiting enzyme in the process of tissue fatty acid intake from serum. Up-regulated LPL led to depressed fat meat percentage and drip loss rate, whereas the meat color and lean meat percentage was increased [35]. Zhu reported that the mRNA abundance of LPL was positively correlated with IMF [36]. In this study, LPL was low expressed in Min pig associated with high IMF, which suggested that LPL had no obvious correlation with IMF. ACSL family members catalyze the formation of long chain acyl-CoA from fatty acid, ATP and CoA, playing an important role in both de novo lipid synthesis and fatty acid catabolism. Research now proves that high expressed ACSL increased intracellular fat deposition [37]. In Min pig, ACSL had a lower expression level compared with LW, which might affect lipid synthesis and fatty acid catabolism at the same time and influence the fat deposition of Min pig. 

Carnitine palmitoyltransferase 1 (CPT1) is the rate-controlling enzyme of mitochondrial fatty acid beta-oxidation. CPT1 influenced on the rate of fatty acid oxidation directly [38]. In the Min_LM, CPT1 had a lower expression level than LW_LM, which indicated that the rate of fatty acid oxidation in Min was weaker than LW. Fat deposition could be affected by fatty acids degradation through fatty acid oxidation, which contributes to the different trait of meat quality between Min and LW pig. 

Fat content is the result of a dynamic balance between fatty acid synthesis and degradation, and multiple factors exist in lipid metabolism which should be comprehensively considered. Although mRNA abundance has been determined in this study, polymorphisms and post-translational modification of DEGs have a similar impact on meat quality. Thus, it warrants further investigation of multiple omics analysis among the genome, transcriptome and Proteome levels. 

### 3.4. Nutritional Metabolism

Amino acid is the main component of protein. The type and content of amino acid in food is an important index to measure its sensory taste [39], as well as the contents of amino acid and carbohydrate [40]. In Min_BF, 4-hydroxyphenylpyruvate dioxygenase and primary-amine oxidase were more expressed, while sarcosine oxidase was less expressed compared with Min_LM. These enzymes play roles in the pathway of phenylalanine metabolism, glycine, serine and threonine metabolism, and tyrosine metabolism. At the same time, glucose transporter 2 (GLUT2) presented in the pathway of carbohydrate digestion and absorption had a lower expression level in Min_BF than in Min_LM. The different expression levels of these key factors which affected amino acid metabolism or carbohydrate digestion and absorption may result in various contents of amino acid and carbohydrate. The contents of amino acid and carbohydrate are the principal contributor to the differences of flavors and odors, or even meat quality between BF and LM of Min pig.

## 4. Materials and Methods

### 4.1. Sample Collection

All the experimental protocols were approved on the 27 December 2016 by the Institutional Animal Care and Use Committee of Northeast Agricultural University, Harbin, China (CAS 235-2014). Pigs were housed in an environmental and dietary controlled swine barn at the Institute of Animal Husbandry Research, Heilongjiang Academy of Agricultural Sciences (Harbin, China). Three Min and three Large White male pigs weighing 86–95 kg were chosen randomly. Pigs were sacrificed followed by muscle sample collection from the middle portion (between the 10th and 12th ribs) of LM. Concurrently, BF samples were collected from the Min pigs. All the samples were frozen in liquid nitrogen immediately and stored at −80 °C until further processing.

### 4.2. RNA Preparation and Sequencing

Total RNA of each muscle sample was extracted from approximately 50 mg of frozen tissue using TRIzol Reagent (Invitrogen Corporation, Carlsbad, CA, USA) following the manufacturer’s instructions. The quality of the RNA samples was checked using an Agilent 2100 Bioanalyzer (Agilent Technologies, Santa Clara, CA, USA). The total RNA of 9 samples were used for sequencing with NEBNext^®^ UltraTM RNA Library Prep kit for Illumina^®^ (NEB, Ipswich, MA, USA); all the standards and procedures were performed following the manufacturer’s protocols. After the quality control using Agilent 2100 Bioanalyzer and ABI StepOnePlus Real-Time PCR System (ABI, Vernon, CA, USA), the library preparations were sequenced on an Illumina Hiseq 4000 platform (Illumina, San Diego, CA, USA) and 100 bp paired-end reads were generated.

### 4.3. RNA-Seq Data Analysis and DEGs Analysis

Clean reads were obtained by removing reads containing adapter or poly-N and low quality reads from raw reads. Clean reads were aligned against NCBI Genome *Sus scrofa* with HISAT2 v2.0.4 (Available online: http://www.ccb.jhu.edu/software/hisat). Then, gene expression was estimated using RSEM v1.3.0 (Available online: http://deweylab.github.io/RSEM/) and FPKM value was calculated. In order to identify DEGs, normalized expression data was analyzed with DEseq2 (Fold Change ≥ 2.00, *p* ≤ 0.05) and PossionDis (Fold Change ≥ 2.00, false discovery rate (FDR) ≤ 0.001). The differentially expressed genes were sorted by the enrichment of GO categories and KEGG database in the Database for Annotation, Visualization and Integrated Discovery (DAVID) Bioinformatics Resources (Available online: http://david.abcc.ncifcrf.gov/). 

### 4.4. Validation of DEGs by Real-Time PCR

To validate the expression level of the DEGs, real-time PCR was performed. Genes in Table 4 were randomly selected for the validation. The first thirteen genes were selected for Group LM, while the last eight genes were selected for Group Min. All primers were designed by Primer Premier 5.0 and synthesized by TsingKe Biological Technology (Beijing, China). Total RNA was extracted as described previously, and reverse transcribed to cDNA using PrimeScriptTM RT reagent Kit (Takara, Japan). Real-time PCR was performed with the UltraSYBR Mixture (CWBIO, Beijing, China) using a StepOne Real-Time PCR system (Applied Biosystems, Foster City, CA, USA). Three replicates were performed for each reaction. Results were expressed by 2^−ΔΔ*C*t^ value and *GAPDH* was chosen as an internal reference.

## 5. Conclusions

In this study, the expressions of 1371 genes differ in the *longissimus dorsi* muscle of Min compared with LW pig, of which 63 pathways was enrichment. In addition, 114 DEGs were in the *biceps femoris* muscle of Min when compared with the *longissimus dorsi* muscle, of which 28 pathways was enrichment. The differences of meat quality between different muscles (determined by breeds or cuts) may be caused by: (1) the IRS1/Akt/FoxO1 signaling pathway induced high expression of slow muscle fibers; (2) AMPK cascade effects induced high IMF; (3) increased fat deposition in the muscle as indicated by the high expressed LXRα and low expressed FABP3, LPL, ACS, CPT1; (4) amino acid composition differences caused by phenylalanine, tyrosine, glycine, serine and threonine metabolism. Collectively, transcriptome analyses provide valuable information for the studies on molecular mechanism of meat quality trait formation, as well as contribute to improving the meat quality of livestock and poultry.

## Figures and Tables

**Table 1:** Number of single-end 100 bp clean reads obtained and percentages of mapped reads per individual.

Group	Individual	Clean Reads	% Mapped Reads	Q20 (%)
LW_LM	LW_LM1	30,149,804	87.49	98.01
	LW_LM2	30,149,790	85.72	98.15
	LW_LM3	30,149,412	86.25	97.36
Min_LM	Min_LM1	30,149,856	85.95	97.81
	Min_LM2	30,149,510	86.74	98.03
	Min_LM3	30,149,878	84.95	97.83
Min_BF	Min_BF1	30,080,960	82.82	97.53
	Min_BF2	30,122,758	81.22	97.64
	Min_BF3	30,455,930	84.11	98.14

**Table 2:** Pathways enriched in Group LM.

Pathway	*p*-Value	*q*-Value
Insulin resistance	1.53 × 10^−10^	4.59 × 10^−8^
Starch and sucrose metabolism	7.09 × 10^−6^	1.06 × 10^−3^
Regulation of actin cytoskeleton	4.09 × 10^−5^	3.96 × 10^−3^
Insulin signaling pathway	5.28 × 10^−5^	3.96 × 10^−3^
PPAR signaling pathway	8.21 × 10^−5^	4.82 × 10^−3^
Regulation of lipolysis in adipocytes	1.07 × 10^−4^	4.82 × 10^−3^
Adipocytokine signaling pathway	1.12 × 10^−4^	4.82 × 10^−3^
Glucagon signaling pathway	1.46 × 10^−4^	4.97 × 10^−3^
Bladder cancer	1.49 × 10^−4^	4.97 × 10^−3^
PI3K-Akt signaling pathway	2.20 × 10^−4^	6.60 × 10^−3^
Pathways in cancer	2.81 × 10^−4^	7.51 × 10^−3^
Complement and coagulation cascades	3.01 × 10^−4^	7.51 × 10^−3^
Platelet activation	3.63 × 10^−4^	7.63 × 10^−3^
MAPK signaling pathway	3.67 × 10^−4^	7.63 × 10^−3^
MicroRNAs in cancer	3.82 × 10^−4^	7.63 × 10^−3^
Circadian rhythm	6.99 × 10^−4^	1.27 × 10^−2^
Amoebiasis	7.21 × 10^−4^	1.27 × 10^−2^
AMPK signaling pathway	9.67 × 10^−4^	1.46 × 10^−2^
Small cell lung cancer	9.72 × 10^−4^	1.46 × 10^−2^
FoxO signaling pathway	9.74 × 10^−4^	1.46 × 10^−2^
Proteoglycans in cancer	1.62 × 10^−3^	2.32 × 10^−2^
Focal adhesion	2.19 × 10^−3^	2.89 × 10^−2^
Riboflavin metabolism	2.22 × 10^−3^	2.89 × 10^−2^
Rap1 signaling pathway	2.36 × 10^−3^	2.95 × 10^−2^
ECM-receptor interaction	2.96 × 10^−3^	3.44 × 10^−2^
Cocaine addiction	2.98 × 10^−3^	3.44 × 10^−2^
Hypertrophic cardiomyopathy (HCM)	3.50 × 10^−3^	3.88 × 10^−2^
Carbohydrate digestion and absorption	4.85 × 10^−3^	5.20 × 10^−2^
Pertussis	6.19 × 10^−3^	6.40 × 10^−2^
Protein digestion and absorption	6.48 × 10^−3^	6.48 × 10^−2^

**Table 3:** Pathways enriched in Group Min.

Pathway	*p*-Value	*q*-Value
Focal adhesion	1.74 × 10^−6^	3.05 × 10^−4^
Pertussis	9.30 × 10^−5^	5.45 × 10^−3^
Protein digestion and absorption	9.58 × 10^−5^	5.45 × 10^−3^
Amoebiasis	1.44 × 10^−4^	5.45 × 10^−3^
ECM-receptor interaction	1.55 × 10^−4^	5.45 × 10^−3^
PI3K-Akt signaling pathway	6.42 × 10^−4^	1.68 × 10^−2^
Prion diseases	7.24 × 10^−4^	1.68 × 10^−2^
AGE-RAGE signaling pathway in diabetic complications	7.65 × 10^−4^	1.68 × 10^−2^
Complement and coagulation cascades	1.10 × 10^−3^	2.14 × 10^−2^
Platelet activation	1.45 × 10^−3^	2.55 × 10^−2^
Staphylococcus aureus infection	2.54 × 10^−3^	4.06 × 10^−2^
Regulation of actin cytoskeleton	3.36 × 10^−3^	4.92 × 10^−2^
Carbohydrate digestion and absorption	5.37 × 10^−3^	7.00 × 10^−2^
Vascular smooth muscle contraction	5.57 × 10^−3^	7.00 × 10^−2^
Salmonella infection	9.99 × 10^−3^	1.17 × 10^−1^
Oxytocin signaling pathway	1.21 × 10^−2^	1.27 × 10^−1^
Phenylalanine metabolism	1.23 × 10^−2^	1.27 × 10^−1^
MicroRNAs in cancer	1.37 × 10^−2^	1.34 × 10^−1^
Butanoate metabolism	1.56 × 10^−2^	1.45 × 10^−1^
Thyroid hormone signaling pathway	2.02 × 10^−2^	1.78 × 10^−1^
Pathogenic Escherichia coli infection	2.20 × 10^−2^	1.84 × 10^−1^
EGFR tyrosine kinase inhibitor resistance	2.42 × 10^−2^	1.86 × 10^−1^
Axon guidance	2.43 × 10^−2^	1.86 × 10^−1^
Leukocyte transendothelial migration	2.77 × 10^−2^	2.03 × 10^−1^
Glycine, serine and threonine metabolism	3.22 × 10^−2^	2.27 × 10^−1^
Tyrosine metabolism	3.46 × 10^−2^	2.34 × 10^−1^
Insulin secretion	3.70 × 10^−2^	2.41 × 10^−1^
Fat digestion and absorption	4.76 × 10^−2^	2.91 × 10^−1^

**Table 4:** Primer sequences for the real-time PCR amplification of the differential expressed genes.

Gene	Primer Sequences (5′-3′)	Product Size (bp)	T_m_ (°C)
*CPT1A*	F: ACAAGCCATAGTCTTAACGAAA; R: GCCAGTCCAGGATAACAAA	198	60
*CPT1B*	F: ACTGTCTGGGCAAACCAAAC; R: CTTCTTGATGAGGCCTTTGC	176	60
*RasGRP3*	F: TAAATCGCAGCCTACCTCCCCT; R:TTGGCAGCTATACTTTCAAAGTCCT	198	60
*IRS1*	F: TGCCTGACCAGCAAGACCATC; R: ATCCACCTGCATCCAAAACTC	168	60
*UCP3*	F: GACGTGGTGAAGGTTCGATT; R: CGAGTTCATGTACCGGGTCT	330	60
*CRYAB*	F: GACCCTCTCACCATTACTTCA; R: CAGCAGGCTTCTCTTCACG	121	60
*PYGM*	F: CCCAGTATGCCAGGGAGAT; R: CTGAGGGATTGCGAACAGA	125	60
*HSPB1*	F: CCTGTCACTTTCGAGGCG; R: AGGTGGGGATGGCTGGT	168	60
*PPARA*	F: CCGAGACCGCAGATCTCAAG; R: GACGAAAGGCGGGTTATTGC	128	60
*PPARGC1A*	F: GATGTGTCGCCTTCTTGTTC; R: CATCCTTTGGGGTCTTTGAG	93	60
*GPX3*	F: GCTTCCCCTGCAACCAATT; R:GGACATACCTGAGAGTGGACAGAA	75	60
*AMD1*	F: TCCACAAGTCAAGTCCTCTAATG; R: CCATGGAGAGGAACGAATCAA	108	60
*ZIC1*	F: CGACCGACGCTTTGCTAATA; R: GTAGGACTTGTCGCACATCTT	97	60
*GAPDH*	F: CTACTCGGGCCTCTTCTGTG; R: GATTCTCCCGATCAGTCAGC	112	60
*PLP1*	F: CTTCCTTTATGGGGCCCTCC; R: ACACACCCGCTCCAAAGAAT	181	60
*NEFM*	F: GAGCAGAACAAGGAGGCCAT; R: TTGGTGCCTCGAACTGACTC	104	60
*ACSM3*	F: AATGGCTCCACCAATCCAGG; R: ACGTTGGTCTTGGCAGTAGC	102	60
*ISLR2*	F: CGTGCACTGAGCTCTTCAGG; R: CGGGGTTCAACTCCTTTTCC	115	60
*ZNF503*	F: CCAAACATGCTCGCAGATCG; R: ATGTCGCTTAGCTTGAGGGG	150	60
*HOXC6*	F: GCCTTTCTCCTGGTGTACTGT; R: TCCTGCCCTGCTCAGAACTAA	193	60
*SIM1*	F: GGCTCTCACCGGCAGTATTT; R: TGAGCCATTACAGCCCAAGG	114	60
*ZIC1*	F: GCCTCCATTCCCTATCCTGC; R: TGAGCGTTTGTGCTTGTTCG	145	60
